# Characterization of a proteomic profile associated with organ dysfunction and mortality of sepsis and septic shock

**DOI:** 10.1371/journal.pone.0278708

**Published:** 2022-12-02

**Authors:** Adolfo Ruiz-Sanmartín, Vicent Ribas, David Suñol, Luis Chiscano-Camón, Clara Palmada, Iván Bajaña, Nieves Larrosa, Juan José González, Núria Canela, Ricard Ferrer, Juan Carlos Ruiz-Rodríguez

**Affiliations:** 1 Department of Intensive Care, Vall d’Hebron University Hospital, Vall d’Hebron Barcelona Hospital Campus, Barcelona, Spain; 2 Shock, Organ Dysfunction and Resuscitation (SODIR) Research Group, Vall d’Hebron Research Institute, Barcelona, Spain; 3 Departament de Medicina, Universitat Autònoma de Barcelona, Barcelona, Spain; 4 Eurecat, Centre Tecnològic de Catalunya, Digital Health Unit, Barcelona, Spain; 5 Department of Clinical Microbiology, Vall d’Hebron University Hospital, Vall d’Hebron Barcelona Hospital Campus, Barcelona, Spain; 6 Department of Genetics and Microbiology, Universitat Autònoma de Barcelona, Barcelona, Spain; 7 CIBERINFEC, ISCIII–CIBER de Enfermedades Infecciosas, Instituto de Salud Carlos III, Madrid, Spain; 8 Eurecat, Centre Tecnològic de Catalunya, Centre for Omic Sciences (COS), Joint Unit URV-EURECAT, Unique Scientific and Technical Infrastructures (ICTS), Reus, Spain; Pacific Northwest National Laboratory, UNITED STATES

## Abstract

**Introduction:**

The search for new biomarkers that allow an early diagnosis in sepsis and predict its evolution has become a necessity in medicine. The objective of this study is to identify, through omics techniques, potential protein biomarkers that are expressed in patients with sepsis and their relationship with organ dysfunction and mortality.

**Methods:**

Prospective, observational and single-center study that included adult patients (≥ 18 years) who were admitted to a tertiary hospital and who met the criteria for sepsis. A mass spectrometry-based approach was used to analyze the plasma proteins in the enrolled subjects. Subsequently, using recursive feature elimination classification and cross-validation with a vector classifier, an association of these proteins with mortality and organ dysfunction was established. The protein-protein interaction network was analyzed with String software.

**Results:**

141 patients were enrolled in this study. Mass spectrometry identified 177 proteins. Of all of them, and by recursive feature elimination, nine proteins (GPX3, APOB, ORM1, SERPINF1, LYZ, C8A, CD14, APOC3 and C1QC) were associated with organ dysfunction (SOFA > 6) with an accuracy of 0.82 ± 0.06, precision of 0.85 ± 0.093, sensitivity 0.81 ± 0.10, specificity 0.84 ± 0.10 and AUC 0.82 ± 0.06. Twenty-two proteins (CLU, LUM, APOL1, SAA1, CLEBC3B, C8A, ITIH4, KNG1, AGT, C7, SAA2, APOH, HRG, AFM, APOE, APOC1, C1S, SERPINC1, IGFALS, KLKB1, CFB and BTD) were associated with mortality with an accuracy of 0.86 ± 0.05, a precision of 0.91 ± 0.05, a sensitivity of 0.91 ± 0.05, a specificity of 0.72 ± 0.17, and an area under the curve (AUC) of 0.81 ± 0.08 with a confidence interval of 95%.

**Conclusion:**

In sepsis there are proteomic patterns associated with organ dysfunction and mortality.

## Introduction

Sepsis is known as the clinical syndrome of life-threatening dysfunction caused by a dysregulated host response to infection. The severity of sepsis varies significantly with the response and the degree of organ dysfunction. Severe cases of sepsis in which hypotension continues despite adequate fluid replacement, are classified as septic shock [1], which has a mortality rate of around 20–40% [2, 3].

For decades, various biomarkers have been detected and studied that, not only allow an early diagnosis of sepsis, but can also predict the evolution and mortality of these patients. Initially, among them, C-reactive protein (CRP), procalcitonin (PCT), interleukin-6 (IL-6), have been postulated as diagnostic and prognostic biomarkers in sepsis, although with limitation. Plasma CRP and IL-6 concentration is positively correlated with the risk of organ dysfunction and death [4, 5]. Both, however, cannot accurately reflect the severity of infection and sepsis because they may increase during minor infection or remains high even after the time course of infection, as well as increasing during an inflammatory response to noninfectious events, trauma, tumorigenesis or surgical interventions [6–8]. PCT is, probably, the marker that best meets the requirements of a biomarker for infection at present and has even been postulated as a prognostic factor for the evolution of sepsis [9] as well as a guide in the duration of antibiotic treatment [10]. However, various factors, including the time of evolution of sepsis or the presence of renal failure, can cause a lost of confidence when its determination is made in isolation [11, 12]. Other biomarkers such as preseptin or pro-adrenomedullin (pro-ADM) have also been postulated as promising biomarkers in sepsis [13, 14].

In recent years, different technologies such as genomics, epigenetics, transcriptomics, proteomics, and metabolomics have been used to investigate the pathogenesis of sepsis. The role of omics technologies in this field is to find biomarkers that differentiate infectious from noninfectious inflammation, to find biomarkers that predict clinical outcome and offer the possibility of therapy for sepsis, and to discover biomarkers that can predict patient response to therapy. Of all of them, proteomics is perhaps the omics methodology that has aroused the most expectations in the field of medicine in general and, in that of infectious diseases, in particular. Its objective is the systematic and large-scale analysis of the proteome (the set of all the proteins expressed by the genome of a cell, tissue, or organ) at a given moment and under certain conditions of time and environment, resulting in biomarker molecules for numerous diseases, since they are involved in most cellular physiological, and pathological processes [15, 16]. Compared to other immunological tests, proteomic is a method that has the advantages of high throughput, sensitivity, and specificity. The development of these techniques has provided important means for studying cellular processes, such as cell signaling, the identification of protein modifications, and the characterization of specific biological markers [16].

This study hypothesizes that there may be proteomic profiles associated with organ dysfunction and sepsis mortality. The objective of this study is to identify potential protein biomarkers that are expressed in patients admitted to a tertiary hospital with a diagnosis of sepsis, their role in the process and their relationship with organ dysfunction and mortality.

## Material and methods

### Study design and ethical approval

This prospective, observational, single-center study enrolled consecutive patients from across hospital departments (emergencies, wards, and Intensive Care Unit) who met the criteria for the activation of the Vall d’Hebron University Hospital intra-hospital sepsis code (ISC) [17] between April and 2016 and January 2018. The study was approved by the Clinical Research Ethics Committee of Vall d’Hebron University Hospital [PR (AG) 11–2016, PR (AG) 336–2016, PR (AG) 210/2017] and written informed consent was obtained from all participants. The study complied in full with the General Data Protection Regulation (GDPR) (Regulation (EU) 2016/679)was performed in accordance with the ethical standards laid down in the 1964 Declaration of Helsinki and its later amendments.

### Inclusion and exclusion criteria

The inclusion criteria followed the Sepsis 2 criteria for the diagnosis of sepsis, established at the time of the study [18]. They included adult patients ≥ 18 years of age presenting with either a suspected or documented infection with the presence of at least one of the two following sets of variables, as outlined by ISC: (1) an acute alteration in the level of consciousness not explained by other clinical conditions, or (2) the presence of hyperthermia (axillary temperature > 38.3°C) or hypothermia (axillary temperature < 36, 0°C), and/or tachycardia (> 110 beats per minute), tachypnea (> 30 breaths per minute) or desaturation (SpO2 <90%), as well as arterial hypotension (systolic blood pressure <90 mmHg or mean arterial pressure < 65 mmHg, or > 40 mmHg decrease in baseline systolic blood pressure). These patients were subsequently classified into 2 independent groups: 1) group of survivors/non-survivors and 2) group of patients according to their organ dysfunction with a SOFA cut-off point > 6. Exclusion criteria included non-adult patients, pregnant women, or patients in whom a blood sample or written informed consent could not be obtained.

### Data collection and biomarker measurements

Patient comorbidities and demographics were subsequently noted upon sepsis code activation, as well as data concerning triage, routine laboratory values, microbiology testing and final clinical diagnosis. Clinical scores (SOFA) were calculated within the first 24h of inclusion of the patient in the study. The worst calculated for each patient was taken as the final value. Of the eligible patients, a venous or arterial blood sample was obtained from all patients at the time of the initial visit as well as a sample for microbiological cultures. PCT [chemiluminescent immunoassay (CLIA)], PCR (immunoturbidimetric test) and lactate determinations (enzymatic color test) were performed on these samples. The samples obtained were frozen at -80°C and stored in a Sepsis Bank of the Vall d’Hebron University Hospital Biobank for later analysis, following the protocols of the clinical laboratories. A subgroup analysis was performed based on mortality and organ dysfunction.

### Protein study

The proteomic study was performed from plasma samples collected in Vacutainer K2E EDTA tubes (Becton Dickinson-Plymouth, United Kingdom) by the Proteomics and Metabolomics Area of the Center for Omic Sciences (COS), a Joint Unit between Rovira I Virgili University and Eurecat (Reus, Spain)

### Protein extraction and quantification

Prior to proteomic analysis, depletion of the seven most abundant plasma proteins (albumin, IgG, antitrypsin, IgA, transferrin, haptoglobin, and fibrinogen) was performed to increase the number of identified/quantified proteins. Therefore, 12 μl of each sample was passed twice through the Agilent Technologies Human-7 Multiple Affinity Removal Spin (MARS) cartridge and flow-through fractions were collected for proteomic analysis following the manufacturer’s protocol. Flow-through fractions were concentrated, and buffer was exchanged to approximately 100 μl of 6 M urea in 50 mM ammonium bicarbonate (ABC) using 5K MWCO spin columns (Agilent 5185–5991).

### Protein digestion and peptide 10-plex TMT labeling

Thirty micrograms of total protein (quantified by Bradford’s method) were reduced with 4mM 1.4-Dithiothreitol (DTT) for 1h at 37°C and alquilated with 8 mM iodoacetamide (IAA) for 30 min at 25°C in the dark. Afterwards, samples were overnight digested (pH 8.0, 37°C) with sequencing-grade trypsin (Promega) at enzyme: protein ratio of 1:50. Digestion was quenched by acidification with 1% (v/v) formic acid and peptides were desalted on Oasis HLB SPE column (Waters) before TMT 10-plex labelling (Thermo Fisher) following manufacturer instructions.

To normalize all samples in the study along the different TMT-multiplexed batches used, a pool containing all the samples was labelled with a TMT-126 tag and included in each TMT batch. The different TMT 10-plex batches were desalted on Oasis HLB SPE columns before the nanoLC-MS analysis.

### nanoLC-(Orbitrap)MS/MS analysis

Labelled and multiplexed peptides were loaded on a trap nano-column (100 μm I.D.; 2cm length; 5μm particle diameter, Thermo Fisher Scientific, San José, CA, USA) and separated onto a C-18 reversed phase (RP) nano-column (75μm I.D.; 15cm length; 3μm particle diameter, Nikkyo Technos Co. LTD, Japan) on an EASY-II nanoLC from Thermo Fisher. The chromatographic separation was performed with a 180 min gradient using Milli-Q water (0.1% formic acid) and acetonitrile (0.1% formic acid) as mobile phase at a flow rate of 300 nL/min.

Mass spectrometry analyses were performed on an LTQ-Orbitrap Velos Pro from Thermo Fisher by an enhanced FT-resolution MS spectrum (R = 30,000 FHMW) followed by a data dependent FT-MS/MS acquisition (R = 15,000 FHMW, 40% HCD) from the most intense ten parent ions with a charge state rejection of one and dynamic exclusion of 0.5 min.

### Protein identification/quantification

Protein identification/quantification was performed on Proteome Discoverer software v.1.4.0.288 (Thermo Fisher). For protein identification, all MS and MS/MS spectra were analyzed using Mascot search engine (v.2.5). Mascot was set up to search SwissProt_2018_03. fasta database (557012 entries), restricting for Human taxonomy (20317 sequences) and assuming trypsin digestion. Two missed cleavages were allowed and an error of 0.02 Da for FT-MS/MS fragmentation mass and 10.0 ppm for a FT-MS parent ion mass were allowed. TMT-10plex was set as quantification modification and oxidation of methionine and acetylation of N-termini were set as dynamic modifications, whereas carbamidometylation of cysteine was set as static modifications. The false discovery rate (FDR) and protein probabilities were calculated by Perclorator. For protein quantification, the ratios between each TMT-label against 126-TMT label were used and quantification results were normalized based on protein median. The results are a ratio of reporter ions abundance and are dimensionless.

## Statistics analysis

Demographic, clinical and laboratory data were reported as mean ± standard deviation (SD) or median with interquartile range (IQR) as appropriate, and categorical variables as numbers and percentages. The statistical analysis was performed using the SPSS 18.0 software (SPSS Inc., Chicago, IL, USA).

In the proteomic study, before any statistical analysis was performed, each protein was standardized and missing values were imputed using the KNN method for proteins that were less than 25% missing. Proteins with major unassignment were censored from the study. The Mann-Whitney U test (p < 0.05) was used to test differences between distributions. The Benjamini-Hochberg procedure was applied to control the false discovery rate (FDR). Statistical analyzes were performed in Python 3.8 using the pandas, sklearn, spicy, and statsmodels libraries.

### Protein selection

Protein selection was performed in three steps. In the first step, the data was divided into training and test sets containing, respectively, 80% and 20% of the data. Any variable containing more than 25% of missing values (65 of 177) was removed. After that, the variables were standardized with a z-score, which was fitted with the train data and applied to both the training and test sets. Missing values were imputed using the k-nearest neighbor (KNN) method, which was fitted to the train data and applied to both the train and the test sets. Finally, recursive feature elimination (RFE) classification was applied in logistic regression with a cross-validated classifier for organ dysfunction or mortality. This method was executed with 10-fold cross validation, adding each result, and finally ranking each protein according to its elimination rate.

In a second step, 60 protein lists were created—the first list containing only the first ranking protein, the second with the two best ranking proteins, and so on—and evaluated with 100-fold cross validation with a support vector classifier (SVC) taking as performance metrics the accuracy, precision, recovery, the harmonic mean of precision and sensitivity (F1 score) and area under the curve (AUC). For each protein list, we report the mean of 100 loops and the spread of the Accuracy, Precision, Recovery, F1 score, and AUC to select the best protein list over which the classification model shall be developed. The significance of the resulting classifier was tested with the permutation test score with the package scikit-learn 1.0.2 implemented in Python.

The impact of the protein on organ dysfunction and mortality was performed through the extraction of the SVC of maximum similarity (that is, we extracted the single SVC closest to the mean of the results obtained in the 100 experiments executed). The coefficients of our SVC were analyzed through their additive Shapley explanations (shape values in summary), in such a way that proteins that presented a positive shap value were associated with higher organ dysfunction. Regarding mortality, negative shap values are associated with higher mortality. The strength of association between the shap value and the outcome (organ dysfunction and mortality) is measured by the magnitude of these shap value.

Protein selection was performed in Python 3.8 using the standard libraries pandas and scikit-learn. The protein-protein interaction network was analyzed with String v 11.0b software (https://string-db.org/).

## Results

### Characteristics of the study population

A total of 141 patients were included in this study. The demographic and clinical data of the patients are shown in Table 1. The most frequent source of infection was urinary 49 (34.8%) followed by respiratory 47 (33.3%) and abdominal 44 (31.2%). All patients had positive cultures. 63 patients (44.68%) had a SOFA > 6 and hospital mortality was 23.4% (33 patients).

**Table 1:** Characteristics of the study population.

Characteristics	Total patients
	(n = 141)
Male, n (%)	85 (60.3)
Age, year (mean ± SD)	63.82 ± 15.56
SOFA score, median (25th, 75th)	6 (5, 8)
Cardiovascular	3 (1–4)
Respiratory	2 (1–3)
Liver	0 (0–1)
Coagulation	0 (0–1)
Renal	1 (0–2)
Neurological	0 (0–1)
SOFA > 6, n (%)	63 (44.68)
Sepsis / Septic shock, n (%)	67 (47.5) / 74 (52.5)
Admitted ICU, n (%)	70 (49.6)
Mechanical ventilation, n (%)	42 (29.8)
Laboratory findings	
Leucocytes (x10E6), (mean ± SD)	13289.35 ± 10795.70
Platelets (x10E6), median (25th, 75th)	175000 (110500, 278500)
Lactate (mmol/L), median (25th, 75th)	2.6 (1.9, 4.2)
CRP (mg/dL), (mean ± SD)	21.8 ± 12.5
PCT (ng/mL), median (25th, 75th)	6.44 (2.02, 25.2)
Infection source, n (%)	
Urinary	49 (34.8)
Respiratory	47 (33.3)
Abdominal	44 (31.2)
Mortality, n (%)	33 (23.4)

### Proteomic study results

Mass spectrometry identified 177 proteins. Of all of them, and by RFE, nine proteins were associated with organ dysfunction (SOFA > 6) with an accuracy of 0.82 ± 0.06, precision of 0.85 ± 0.093, sensitivity 0.81 ± 0.10, specificity 0.84 ± 0.10 and AUC 0.82 ± 0.06. Twenty-two proteins were associated with mortality with an accuracy of 0.86 ± 0.05, a precision of 0.91 ± 0.05, a sensitivity of 0.91 ± 0.05, a specificity of 0.72 ± 0.17, and an area under the curve (AUC) of 0.81 ± 0.08 with a confidence interval of 95%. The analyzed proteins are presented in Table 2.

**Table 2:** Proteins analyzed and their relationship with mortality /organ dysfunction.

PROTEINS ASSOCIATED TO MORTALITY	PROTEINS ASSOCIATED TO ORGAN DYSFUNCTION
**CLU**- Clusterin	**GPX3**- Glutathione peroxidase 3
**LUM**- Lumican	**APOB**- Apolipoprotein B-100
**APOL1**- Apolipoprotein L-1	**ORM1**- Alpha-1-acid glycoprotein 1
**SAA1**- Serum amyloid A-1 protein	**SERPINF1**- Pigment epithelium-derived factor
**CLEBC3B**- Tetranectin	**LYZ**- Lysozyme C
**C8A**- Complement component C8 alpha chain	**C8A**- Complement component C8 alpha chain
**ITIH4**- Inter-alpha-trypsin inhibitor heavy chain family member 4	**CD14**- Monocyte differentiation antigen CD14
**KNG1**- Kininogen-1	**APOC3**- Apolipoprotein C-III
**AGT**- Angiotensinogen	**C1QC**- Complement C1q subcomponent subunit C
**C7**- Complement component C7	
**SAA2**- Serum amyloid A-2 protein	
**APOH**- Beta-2-glicoproteína 1	
**HRG**- Histidine-rich glycoprotein	
**AFM**- Afamin	
**APOE**- Apolipoprotein E	
**APOC1**- Apolipoprotein C-1	
**C1S**- Complement C1s subcomponent	
**SERPINC1**- Antithrombin-III	
**IGFALS**- Insulin-like growth factor-binding protein complex acid labile subunit	
**KLKB1**- Plasma Kallikrein	
**CFB**- Complement Factor B	
**BTD**- Biotinidase	

**Fig 1:**
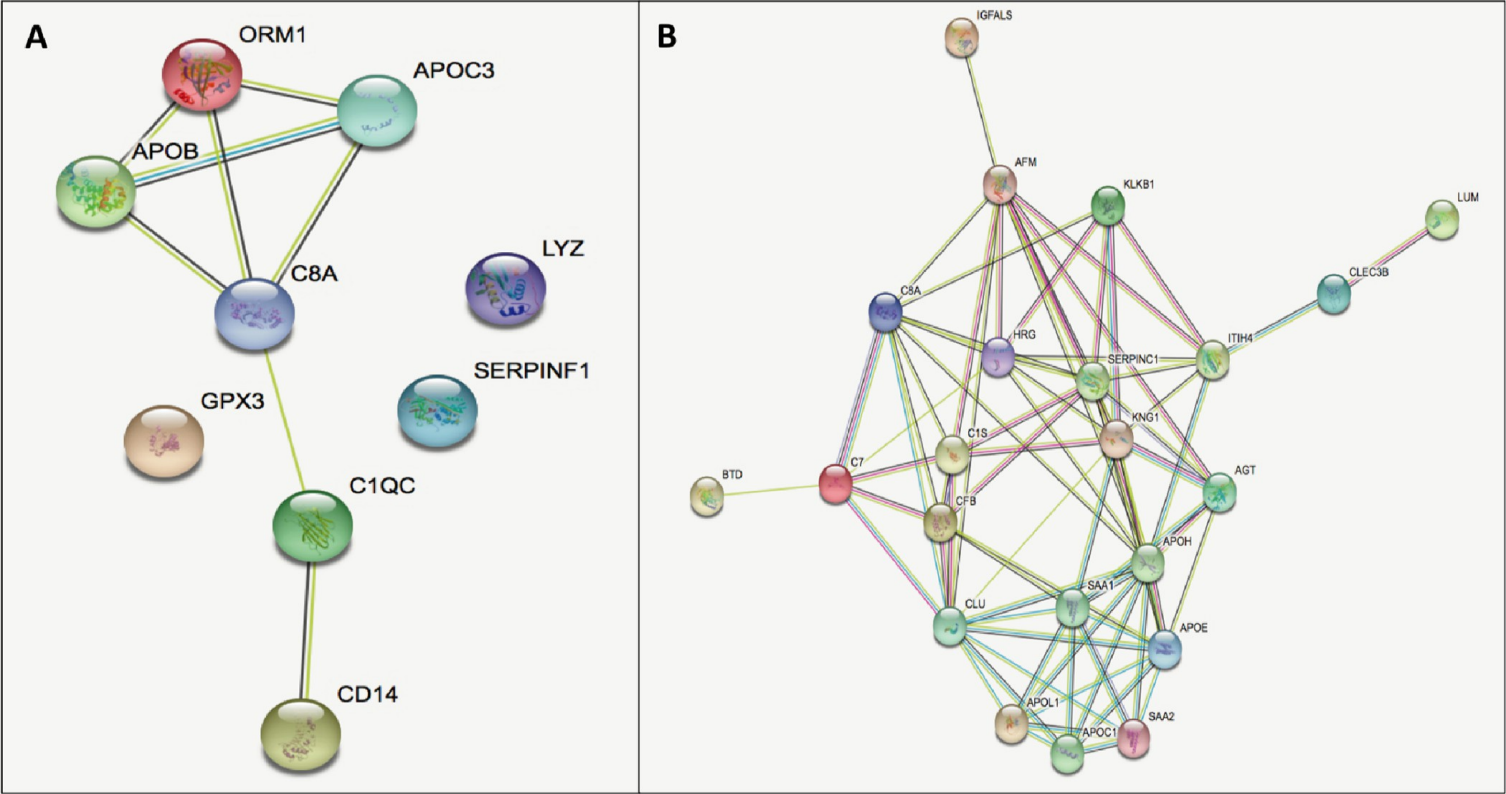
Relationship between proteins (strings): A) organ dysfunction, B) Mortality (https://string-db.org/).

**Fig 2:**
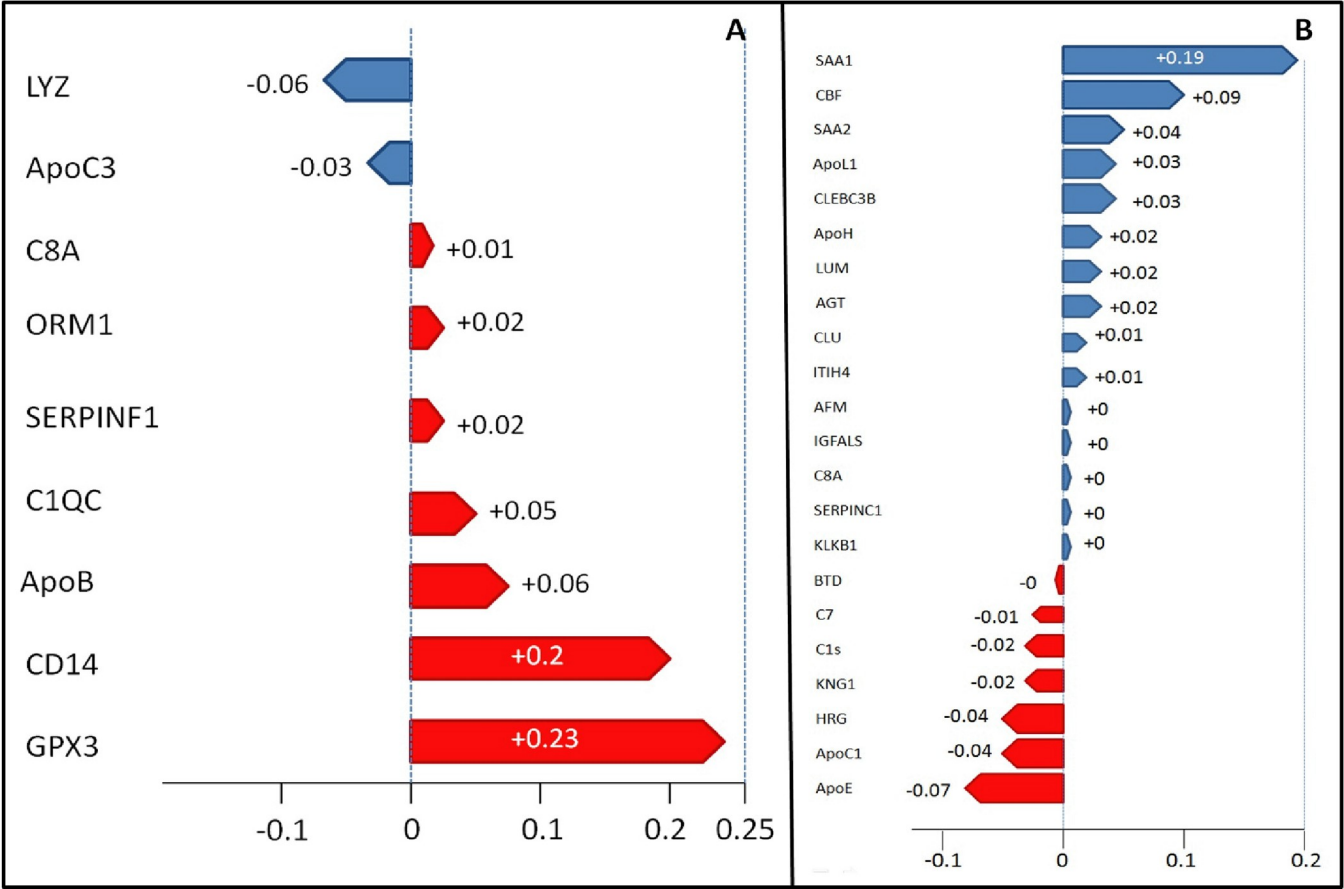
Shap values graphics. A) blue = proteins expressed in patients with SOFA ≤ 6, red = proteins expressed in patients with SOFA > 6. B) blue = proteins expressed in patients survivors, red = proteins expressed in patients non-survivors.

Of the proteins associated with organ dysfunction (p-value = 0.001); two act on complement activation (C1QC and C8a), two on lipoprotein metabolism (APOB and APOC3), three on the inflammatory response (CD1 4, ORM1 and LYZ), three on the regulation of proteolysis (C1QC, C8a and SERPINF1), six on the innate immune system response (CD14, C1QC, C8a, APOB, ORM1 and LYZ) and one has other functions (GPX3). The relationship between these proteins is presented in Fig 1. By applying the SVC model and analyzing their values additively, it was observed that the presence of 7 proteins (GPX3, CD14, APOB, C1QC, SERPINF1, ORM and C8A) had a positive shap value, indicating association with higher organ dysfunction, while two proteins (APOC3 and LYZ) had a negative shap value. The presence of proteins GPX3 (+0.23), CD14 (+0.2) and APOB (+0.06) were associated with high organ dysfunction, while proteins APOC3 (-0.06) and LYZ (-0.03) were associated with lower organ dysfunction. (Fig 2).

Of the proteins associated with mortality (p-value = 0.001), thirteen participate in the regulation of proteolysis (CLEC3B, ITIH4, KLKB1, HRG, KNG1, C8a, SERPINC1, CLU, C7, C1s, AGT, APOE and CFB), nine in the regulation inflammatory response (KLKB1, C8a, CLU, C7, C1s, AGT, APOE, CFB and SAA1), ten in the immune response (HRG, KNG1, APOL1, SERPINC1, C8a, C7, C1s, CLU, CFB and SAA1), five in lipid transport (APOH, APOL1, APOC1, CLU and APOE), five in complement activation (C8a, C7, C1s, CFB and CLU), eight in hemostasis (CLEC3B, ITIH4, KLKB1, HRG, KNG1, APOH, SERPINC1 and CLU) and five fulfill other functions (LUM, IGFALS, AFM, BTD and SAA2). The relationship between these proteins is also presented in Fig 1. Applying the same SVC model and shap value analysis, it was observed that, of these proteins, fifteen of them (SAA1, CFB, SAA2, APOL1, CLEC3B, APOH, LUM, AGT, CLU, ITIHT4, AFM, IGFALS, C8A, SERPINC1 and KLB1), had a positive shap value, indicating high association with lower mortality, while seven proteins (APOE, APOC1, HRG, KNG, C1s, C7 and BTD) had a negative shap value, indicating stronger association with higher mortality. Likewise, it was observed that the presence of SAA1 (+0.19), CFB (+0.09) and SAA2 (+0.04) proteins were associated with lower mortality while APOE proteins (-0.07), APOC1 (-0.04) and HRG (-0.04) were associated in patients with higher mortality. (Fig 2).

## Discussion

This study demonstrates the existence of proteomic patterns associated with organ dysfunction and sepsis mortality. A set of nine proteins were associated with organ dysfunction, and seven of them were found predominantly in those patients with upper organ dysfunction (SOFA > 6), while two proteins were found in patients with lower organ dysfunction. In addition, a set of twenty-two proteins that may play a role in the mortality of septic patients, of which thirteen proteins are associated with lower mortality while nine of them have a higher expression in non-surviving patients. The presence and predominance of some over others could tip the balance of the result of evolution. In addition, the different proteins observed in this study participate in different physiological metabolic pathways, which represents sepsis as a very complex process in which various biological processes are involved. This is a first step to evaluate the possible prognostic value of these biomarkers and will allow advancing in the therapeutic strategy that will improve the survival of these patients.

Sepsis involves an extensive systemic inflammatory response that causes the release of cytokines and chemokines. In this setting, proteomics provides an analysis of the expression, location, function, and interaction of proteomes. Compared with other immunological tests, proteomics is a novel method that has the advantages of high throughput, sensitivity, and specificity, which is why it has been widely used in various fields of medicine such as oncology, neurology, and hematology [19–21].

### Apolipoproteins

The role of lipid metabolism in sepsis is well known. Although hypercholesterolemia has been associated with different cardiovascular diseases, hypocholesterolemia has been correlated with the appearance of infections and mortality in older adults [22, 23]. In our study, we show that some apolipoproteins are detrimental in the evolution and outcome of sepsis, while others play the opposite role. In this study we have observed high expression of ApoC3 in patients with less organ dysfunction. ApoC3 acts as the activation of the NLRP3 inflammasome present in human monocytes, which through a complex enzymatic sequence results in the production of interleukin 1β that induces pyroptosis, an inflammatory necrotic cell death [24]. A study evaluating changes in serum proteome between sepsis and non-sepsis groups found that a downregulation of ApoC3 presented good discrimination properties between the two study groups. Patients with sepsis had lower ApoC3 expression than non-septic patients [25]. On the contrary, we have observed that ApoB (with a shap value +0.06) was associated with higher organ dysfunction. Kumaraswamy et al, in a study whose objective was to study the association of ApoM with the severity of sepsis, observed that ApoB behaved as an acute negative phase protein. The largest decreases were seen in patients with severe sepsis with shock, followed by the severe sepsis without shock group and non-infectious systemic inflammatory response syndrome. Thus, like our results, the most dramatic decreases were seen in critically ill patients. In addition, they were correlated with lower levels of ApoA1, HDL, and LDL cholesterol [26].

We have also observed that ApoC1 and ApoE had a stronger association with non-surviving patients. In most of the studies carried out, it has been observed that, in septic patients, serum levels of ApoC1 are lower, which perhaps suggests a possible protective effect. ApoC1 has been shown to provide protection against Klebsiella pneumoniae-induced pneumonia by increasing the early immune response and preventing lethality in mice [27]. In addition, Sharma et al [28] observed that when comparing septic patients with healthy controls, lipid metabolism was the main altered function with downregulated proteins, such as ApoB, ApoC1 and ApoL1 among others. Contrary to this, in other studies, the measured plasma levels of ApoC1 were positively correlated with the proinflammatory response of patients undergoing extracorporeal circulation and who presented endotoxemia during reperfusion [29]. Therefore, ApoC1 regulates, in a complex way, the activity of the enzymes and receptors involved in the metabolism of VLDL and HDL and, then, the global effect on the plasma lipid profile is the result of a subtle control resulting from multiple effects seemingly opposite.

ApoE is the main apolipoprotein of LDL. In this study, ApoE levels were elevated in septic patients who did not survive. In neurodegeneration studies, the presence of endotoxins from gram-negative bacteria was found to bind directly to ApoE, contributing to neuroinflammation and thus neuronal damage [30]. In a study in rats, in which ApoE was injected serially after cecal ligation and puncture, it was shown that 48 h after injections, septic mortality increased in a dose-dependent manner [31]. Other studies in murine models have observed changes in lipoprotein composition in which ApoA-1 and ApoC-1 are decreased while ApoE levels are increased. These changes in the composition of lipids and apolipoproteins accelerate the terminal catabolism of HDL and, therefore, the appearance of hypocholesterolemia [32, 33]. On the other hand, ApoH has been shown to exert a protective role in sepsis as a scavenger of lipopolysaccharides in Gram negatives [34]. In our study, ApoH has turned out to be a lipoprotein with a protective role in mortality. Schrijver et al [35] observed that ApoH levels were significantly lower in septic patients, especially in patients with septic shock, although they found no significant differences between survivors and non-survivors.

### Proteolysis

Proteolysis is an important process in sepsis. In human and murine experiments, increased proteolysis is associated with organ dysfunction and mortality [36, 37]. Most of the proteins detected, in this study, play an important role in proteolysis and their effects on organ dysfunction and mortality are disparate. The role of antithrombin 3 (SERPINF1) as an inhibitor of serine proteases and its natural anticoagulant effect has been known for decades [38]. It has also been shown to exert anti-inflammatory effects during sepsis [39]. Kuroda et al [40] showed that the administration of recombinant antithrombin 3 in septic patients with significant organ dysfunction improved the results of disseminated vascular coagulation (DIC) and improved the score on the SOFA scale, demonstrating a protective role of this protein. ITIH4 (H4 heavy chain inter-alpha-trypsin inhibitor) has been postulated as a biomarker in sepsis [41]. This protein, along with the rest of the inter-alpha-trypsins, is, like antithrombin 3, a family of serine protease inhibitors of hepatic origin with an important role in inflammation [42]. Furthermore, low levels of ITIH4 have been observed in patients with DIC [43], which means that a decrease in ITIH4 levels induces a procoagulant state that can lead to increased organ dysfunction and mortality. Unlike our study, there are others in which there is a positive correlation between ITIH4 levels and the severity of the infection [44, 45].

CLEC3B or tetranectin (TN) is the one that plays a more important role in our study. This plasma protein is known to be secreted by myeloid cells. Its expression is greater in the lungs, although it is also present in the bloodstream of humans. The biological roles of TN are not fully understood, although it has been implicated in multiple bone disorders and wound healing [46]. In this study, TN showed a protective effect against mortality. Chen et al [47] found that sepsis patients showed much lower blood levels of TN compared to healthy controls. The authors found that removing tetranectin in mice aggravated severe inflammation, lung damage, and other features of fatal sepsis, but supplementing mice with tetranectin reduced organ damage and amplified the survival. Recently, low levels of histidine-rich glycoprotein (HRG) have been shown to be associated with increased mortality in septic patients, which could be an important prognostic biomarker [48]. In this study, HGR has a strong association with mortality (with a shap value of -0.04), as well as Kininogen1 (KNG1) (-0.02). The role played by KNG1, as a proinflammatory cytokine, in accelerating the inflammatory process is also known [49]. Hu et al [50], in a study on the effects of sulfentanil in sepsis lung injury concluded that this drug can alleviate inflammation and oxidative stress in sepsis-induced acute lung injury by negatively regulating the expression of KNG1.

### Complement

Another group of proteins that plays an important role in sepsis is complement (CS), the main component of the innate immune system against pathogens [51]. In this study, two proteins were associated with organ dysfunction (C1Qc and C8a) having a significant association with higher organ dysfunction and five with mortality, three [Complement Factor B (CFB), clusterin (CLU) and C8a] with a protective role and two (C7, C1s) with a deleterious role. Petersen et al found that patients with deficiencies in components C6, C7 and C8 predisposed to greater meningococcal infection [52]. Keizer et al, contrary to our results, stated that patients with C7 deficiency had less protection after being vaccinated against meningococcus due to lower production of anticapsular antibodies [53]. In upregulation studies in Alzheimer’s disease and many therapy-resistant cancers, low CLU levels have also been associated with increased meningococcal infection [54, 55]. CFB works in the alternative pathway to activate and amplify the complement system [56]. A study conducted in mice with septic shock indicated that the absence of CFB conferred a protective effect, with better survival and cardiac function and less markedly attenuated acute kidney injury [57]. In contrast, other studies have indicated that the alternative complement pathway is essential in the fight against infection and is activated in clinical situations of septic shock. Clinical studies showed that the active CFB fragment was significantly increased in patients with septic shock [58, 59]. In our study, a strong association between C1QC and organ dysfunction is evidenced. In a recent study, Li et al [60] found that C1QC levels were significantly reduced in the serum of sepsis patients. This decrease was associated with a high AUC not only with higher mortality, but also with a higher SOFA score with an AUC for sepsis prognosis, higher SOFA and mortality.

### Other proteins

We have observed in our patients other proteins with different functions. Glutathione peroxidase-3 (GPX3) was the protein that presents the strongest association with higher organ dysfunction. This molecule is known as a key selenoprotein with antioxidant properties. Selenium and GPx-3 deficiency have been associated with sepsis [61]. Some authors have observed that early decreases in GPX3 are associated with inflammatory response syndrome (SIRS) and organ dysfunction [62]. Adjuvant treatment of patients with high doses of sodium selenite has even been shown to reduce the mortality rate in patients with severe sepsis or septic shock [63].

We have also observed the important protective role of SAA1 and SAA2 in mortality. But contrary to our results, it has been observed that high levels of these two proteins have been associated with a greater appearance of sepsis [64] and their downregulation improves intestinal injury in mice [65]. Although the authors have not been able to find the relationship of these proteins with mortality.

### Limitations

This study has several limitations. First of all, only patients with sepsis have been included in this study and it has not been compared with patients with non-infectious systemic inflammatory response syndrome. Therefore, we do not know if the findings can also be observed in this type of patient and not, specifically, in the inflammatory response induced by the infection. Second, the design of this study only allows us to know that the hyperexpression of a protein is associated with organ dysfunction or mortality, but we do not know not only the concentration of each protein, but also the role that this protein plays in each group. In other words, we know that a protein is hyperexpressed, for example in high organ dysfunction, but we do not know if its presence is deleterious or, on the contrary, a compensatory mechanism that tries to counteract said organic dysfunction. A multicenter study should be carried out with more patients in which not only what proteins are related to the described objectives are observed, but also a metabolic study of the same to be able to specify which are positively associated with organ dysfunction and which with the mortality.

## Conclusion

In sepsis there are proteomic patterns associated with organ dysfunction and mortality. Advances in knowledge of the proteins changes associated with organ dysfunction and sepsis mortality may allow the identification of new therapeutic targets in the future.
